# TRACK-CF prospective cohort study: Understanding early cystic fibrosis lung disease

**DOI:** 10.3389/fmed.2022.1034290

**Published:** 2023-01-06

**Authors:** Eva Steinke, Olaf Sommerburg, Simon Y. Graeber, Cornelia Joachim, Christiane Labitzke, Gyde Nissen, Isabell Ricklefs, Isa Rudolf, Matthias V. Kopp, Anna-Maria Dittrich, Marcus A. Mall, Mirjam Stahl

**Affiliations:** ^1^Department of Pediatric Respiratory Medicine, Immunology and Critical Care Medicine, Charité–Universitätsmedizin Berlin, Berlin, Germany; ^2^German Center for Lung Research (DZL), Associated Partner Site, Berlin, Germany; ^3^Berlin Institute of Health (BIH) at Charité, Berlin, Germany; ^4^Division of Pediatric Pulmonology and Allergy and Cystic Fibrosis Center, Department of Translational Pulmonology, University of Heidelberg, Heidelberg, Germany; ^5^Translational Lung Research Center Heidelberg (TLRC), German Center for Lung Research (DZL), Heidelberg, Germany; ^6^Division of Pediatric Pneumology and Allergology, University of Lübeck, Lübeck, Germany; ^7^Airway Research Center North (ARCN), German Center for Lung Research (DZL), Lübeck, Germany; ^8^Department of Pediatric Pneumology, Allergology and Neonatology, Hannover Medical School, Hannover, Germany; ^9^Biomedical Research in Endstage and Obstructive Lung Disease (BREATH), German Center for Lung Research (DZL), Hannover, Germany; ^10^Division of Respiratory Medicine, Department of Pediatrics, University Children's Hospital, Inselspital, University of Bern, Bern, Switzerland

**Keywords:** cystic fibrosis, early lung disease, non-invasive monitoring, magnetic resonance imaging (MRI), multiple-breath washout (MBW), risk factors in cystic fibrosis, biomarkers in cystic fibrosis

## Abstract

**Background:**

Lung disease as major cause for morbidity in patients with cystic fibrosis (CF) starts early in life. Its large phenotypic heterogeneity is partially explained by the genotype but other contributing factors are not well delineated. The close relationship between mucus, inflammation and infection, drives morpho-functional alterations already early in pediatric CF disease, The TRACK-CF cohort has been established to gain insight to disease onset and progression, assessed by lung function testing and imaging to capture morpho-functional changes and to associate these with risk and protective factors, which contribute to the variation of the CF lung disease progression.

**Methods and design:**

TRACK-CF is a prospective, longitudinal, observational cohort study following patients with CF from newborn screening or clinical diagnosis throughout childhood. The study protocol includes monthly telephone interviews, quarterly visits with microbiological sampling and multiple-breath washout and as well as a yearly chest magnetic resonance imaging. A parallel biobank has been set up to enable the translation from the deeply phenotyped cohort to the validation of relevant biomarkers. The main goal is to determine influencing factors by the combined analysis of clinical information and biomaterials. Primary endpoints are the lung clearance index by multiple breath washout and semi-quantitative magnetic resonance imaging scores. The frequency of pulmonary exacerbations, infection with pro-inflammatory pathogens and anthropometric data are defined as secondary endpoints.

**Discussion:**

This extensive cohort includes children after diagnosis with comprehensive monitoring throughout childhood. The unique composition and the use of validated, sensitive methods with the attached biobank bears the potential to decisively advance the understanding of early CF lung disease.

**Ethics and trial registration:**

The study protocol was approved by the Ethics Committees of the University of Heidelberg (approval S-211/2011) and each participating site and is registered at clinicaltrials.gov (NCT02270476).

## Background

Cystic fibrosis (CF) lung disease is the main cause of mortality and morbidity in patients with CF for whom the emergence of effective mucolytic, anti-infective and causal cystic fibrosis transmembrane conductance regulator (CFTR) modulating therapies has markedly increased disease prospects (1, 2). The accumulation of viscous mucus facilitates neutrophilic inflammation, which propels the development of structural lung disease (3, 4). Inflammation, initially sterile, later impacted upon by bacteria, entails oxidative stress and a vicious cycle of increased mucus, which promotes inflammation, infection and dysbiosis (5). The lung microbiome changes within the first years of life with an increasing detection of pathogens that in turn promote lung damage (6–8). Investigation of newborn screening (NBS) cohorts revealed that CF lung disease starts soon after birth with airway mucus plugging and airtrapping, structural alterations such as bronchial wall thickening, bronchial dilatation, and bronchiectasis and an impairment in lung function in asymptomatic infants already at the age of 3 months (9–11). Repeated investigations through early childhood demonstrate progression of structural and functional changes in preschool children with CF (12, 13).

The introduction of NBS for CF has advanced detection and early treatment initiation in infants with CF (14, 15). A positive risk-benefit assessment especially regarding nutritional outcomes and growth as well as a reduced hospitalization rate has been demonstrated (16–18). Similarly, studies reporting comparison with historic controls have shown an advantage in survival of screened children and have supported the initiation of CF NBS in many countries (19, 20). In Germany, CF NBS has been implemented nationwide in 2016 (21) but regional screening programs, including a pilot study in Southwest Germany starting in 2008, had been introduced earlier (22). The relevance of the NBS is undisputed bearing in mind the early onset of pulmonary alterations and the narrow window of opportunity to prevent irreversible changes by means of early interventions. The interest in the development and progression of the early phase of CF lung disease has therefore risen and novel methods and study endpoints now facilitate the investigation of its pathogenesis (23, 24).

Spirometry used in older patients to investigate lung function, is not feasible in infants and young preschool children, who are not sufficiently cooperative to perform forced breathing manoeuvers. Furthermore, several studies have demonstrated a lack of sensitivity of spirometric parameters for early pulmonary alterations (25, 26). Multiple-breath washout (MBW), a lung function test performed in quiet tidal breathing to investigate ventilation homogeneity, has thus gained importance, as it is sensitive and feasible from infancy onwards (27). Its principal read-out parameter, the lung clearance index 2.5% (LCI_2.5_), detects progression of CF lung disease (13, 28). It furthermore correlates with infection with *Pseudomonas aeruginosa* and airway inflammation and serves as predictor for later spirometry results and survival of patients with CF (13, 29–31).

Computed tomography (CT) is mainly used to examine lung structure due to its high spatial resolution, short investigation time and ubiquitous availability. Despite first CT abnormalities in infants, correlations with findings in MBW were only present from later childhood on (9, 10, 32–34). With respect to the increasing life expectancy of patients with CF, an alternative radiation-free imaging tool is essential (35). Magnetic resonance imaging (MRI) is utilized to examine the pediatric lung with a sensitivity to detect early CF lung disease that is comparable to CT (36–38). In contrast to CT, chest MRI allows a combined analysis of morphological and functional properties with lung perfusion as a surrogate parameter for ventilation deficits (39, 40).

The broad phenotypic variability of lung disease is already present in children with CF. However, differences in onset and progression of lung disease in early childhood, as well as risk factors and biomarkers to predict the course of CF lung disease remain poorly understood (41, 42). Therefore, we set up a prospective multicenter longitudinal study of early CF lung disease, TRACK-CF (clinicaltrials.gov: NCT02270476). This study uniquely incorporates MBW and MRI and an attached biobank in a prospective, comprehensive observational real-world setting in children diagnosed following CF NBS or clinical symptoms. Based on complementary information provided by MRI and MBW and the potential of molecular studies in biological samples, it aims to gain insight into the disease-promoting vs. protective mechanisms within the German Center for Lung Research (Deutsches Zentrum für Lungenforschung, DZL).

## Study design and methods

### Localization / Funding

The TRACK-CF study cohort was initiated at the participating CF centers of the University Hospitals of Heidelberg, Hannover, Luebeck and Berlin, members of the German Center for Lung Research (DZL). The study protocol was approved by the Ethics Committees of the University of Heidelberg (approval S-211/2011) and each participating site and is registered at clinicaltrials.gov (NCT02270476).

### Cohort description

Informed written consent is obtained from all parents or legal guardians of participants. We recruited infants and pre-school children based on a positive CF NBS result from a regional (before comprehensive introduction of CF NBS in September 2016) or national (after comprehensive introduction of CF NBS in Germany) CF NBS program, or clinical symptoms suggestive of CF (22, 43). The German CF NBS protocol is based on an IRT/PAP/DNA protocol including an IRT-dependent safety net strategy (22).

For analysis, study participants are assigned to one of three groups according to the mode and time of diagnosis: i) diagnosis based on CF newborn screening (NBS); ii) early clinical diagnosis (ECD) within the first 4 months of life; and iii) late clinical diagnosis (LCD) after the first 4 months of life. The NBS group consists of children diagnosed with CF following a positive CF NBS test result and confirmation diagnostics (see below “in-/exclusion criteria”). Asymptomatic infants with a prenatal diagnosis or an early diagnosis of CF due to a positive family history are similarly assigned to the NBS group. CF-typical symptoms leading to a clinical diagnosis and attribution to the ECD or LCD group are meconium ileus, failure to thrive, respiratory symptoms and infections or gastrointestinal symptoms.

### In-/exclusion criteria

The diagnosis of CF is based on internationally recognized criteria: i) a sweat chloride concentration ≥ 60 mmol/l or ii) two CF-causing mutations in the *CFTR* gene or iii) impaired CFTR function as determined from transepithelial bioelectric measurements of intestinal current measurements (ICM) (44, 45). Prematurity <30th gestational week as well as a prolonged period of mechanical ventilation within the first 3 months of life and other chronic lung diseases such as bronchopulmonary dysplasia are defined as exclusion criteria. Moreover, previous major surgeries except for meconium ileus and intestinal atresia as well as other major organ dysfunctions not related to CF lead to exclusion. As MRI is one key outcome measure, in case of claustrophobia or known adverse reactions to sedatives used, the child is not eligible.

### Visit schedule

**Figure 1:**
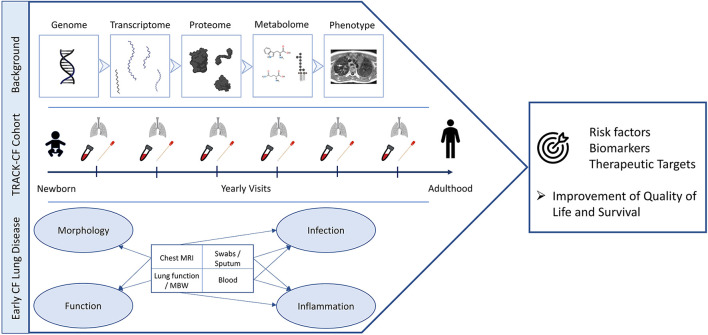
TRACK-CF summary. The strategy to examine the different stages of CF lung disease development (“Background”) is presented on top. The study endpoints and regularly performed examinations from birth to adulthood are displayed in the center and the endpoints and covered aspects of early CF lung disease are shown at the bottom of the figure. The overall aims are to improve the quality of life and the survival through the identification of risk factors, biomarkers and novel therapeutic targets (right). This figure was created with BioRender.com.

**Figure 2:**
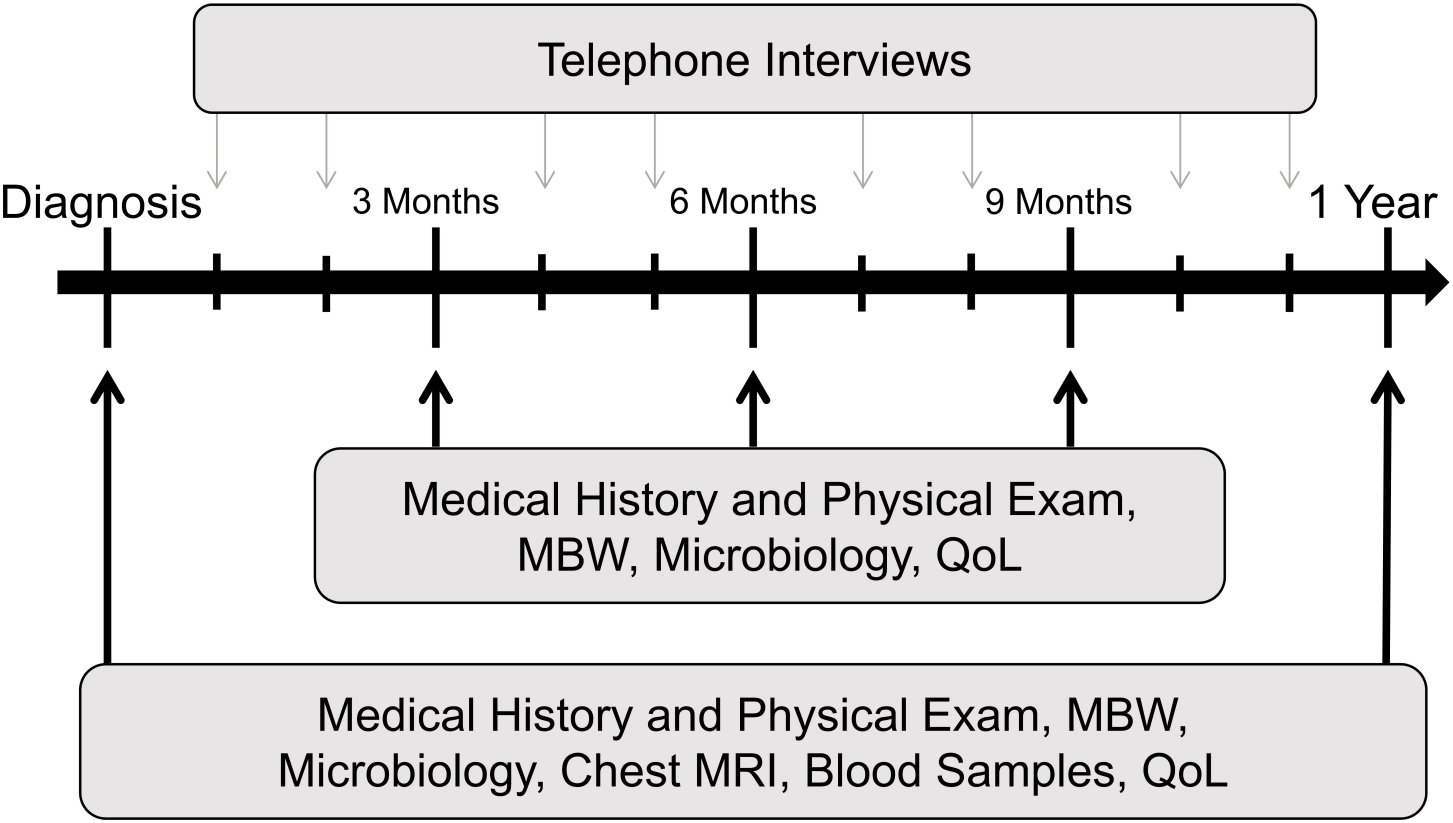
TRACK-CF study design. Exemplary scheme for the first year following diagnosis. Black arrows indicate quarterly routine visits at the CF center; gray arrows indicate study visits performed as telephone interviews in months without a visit to the CF center. This assessment routine is repeated each year. CF, cystic fibrosis; QoL, quality of life questionnaire; MBW, multiple-breath washout; MRI, magnetic resonance imaging.

To minimize the burden for participants and their families, study visits are combined with routine visits at the CF center. Starting from diagnosis, children are followed at the specialized CF center and usually present quarterly for clinical assessments, lung function tests, microbiological swabs and quality of life (QoL) evaluation (Figure 1). The annual check-up furthermore includes blood withdrawal and chest imaging by MRI (Figure 2). Microbiological material such as nose and throat swabs or sputum are similarly taken quarterly as part of the clinical routine. All investigations besides the QoL assessments are part of the clinical routine for all patients with CF. An associated biobank has been established for samples of blood (serum, EDTA, Paxgene Tube), stool, urine, and airway secretion samples (nose swabs, nasal lavage fluids, throat swabs, sputum). CF treatment at all centers complies with the European Practice Guidelines (46, 47). To obtain close monitoring, TRACK-CF study participants are additionally contacted *via* monthly telephone interviews to acquire information on symptoms and the child's general health as well as potential changes in treatment (Table 1).

**Table 1:** Questionnaire and timepoints.

	**Baseline**	**Quarterly visit**	**Annual check-up**	**Monthly telephone interview**
Informed consent	X			
Medical history	X			
Reason for visit (acute / elective)	X	X	X	
Symptoms:				
Cough^*^	X	X	X	X
Sputum^*^	X	X	X	X
Respiratory infection^*^	X	X	X	X
Fever (≥38°C)^*^	X	X	X	X
Dyspnea^*^	X	X	X	X
Activity^*^	X	X	X	X
Weight / appetite^*^	X	X	X	X
Changes in sinonasal symptoms^*^	X	X	X	X
Changes in gastrointestinal symptoms^*^	X	X	X	X
Hospitalizations	X	X	X	X
Medication:				
Antibiotics^*^	X	X	X	X
Inhalations	X	X	X	X
Gastrointestinal	X	X	X	X
CFTR Modulators	X	X	X	X
CFQ-R	X	X	X	
CF-typical complications	X	X	X	

CFTR, Cystic Fibrosis Transmembrane Conductance Regulator; CFQ-R, Cystic Fibrosis Questionnaire-Revised.

* these symptoms are criteria to define an event as pulmonary exacerbation.

## Data measurements

### Medical history by questionnaires/telephone interview

At baseline, the previous medical history focusses on the CF diagnostic procedure. Possible prenatal screening results, sweat test results as well as the family history for CF are included. Similarly, anthropometry at birth and preceding CF-typical symptoms and in- and exclusion criteria are obtained (Table 1).

TRACK-CF questionnaires are completed by a medical professional in cooperation with the parent and child. The same questionnaire is used for telephone interviews, at every study visit and an extended version in case of an annual check-up (Table 1). Telephone interviews are performed monthly by an experienced study nurse with a focus on symptoms within the preceding 4 weeks and ongoing therapy.

### Pulmonary exacerbations

PEx are defined according to modified Fuchs criteria (48). Modification concerns the application of the antibiotic used to treat the PEx, accepting oral administration and inhalation besides intravenous injection. 4 out of 12 criteria including a newly initiated antibiotic treatment have to be met such as cough, sputum, fever, malaise, sinunasal symptoms and weight loss (48).

### Anthropometry

At every quarterly visit, children are examined by an experienced pediatrician with a documentation of anthropometric data to assess growth. Thriving is determined by the standard deviation score (SDS) compared to the German pediatric reference population with normal growth defined as±1.64 SD (49, 50).

### Microbiology

Infections with known proinflammatory pathogens play a crucial role in CF lung disease development and are associated with airway remodeling and destruction (51, 52). Especially *Pseudomonas aeruginosa, Staphylococcus aureus, Haemophilus influenzae, Streptococcus pneumoniae* and *Aspergillus species* have been identified as relevant pathogens in children with CF (51). Quarterly nose and throat swabs or sputum samples, if producible, are examined for diagnostic purposes and to initiate antibiotic treatment where appropriate. Infection status is documented for each pathogen. *Pseudomonas aeruginosa* status is determined based on previous microbiological swabs and antibodies against the pathogen's proteins alkaline protease, elastase and exotoxin A. The status *Pseudomonas aeruginosa* negative is defined as no detection of *Pseudomonas aeruginosa* for at least a year without antibiotic treatment against this pathogen, intermittent *Pseudomonas aeruginosa* infection is specified as a detection of *Pseudomonas aeruginosa* in <50% of the swabs within the previous 12 months. Chronic *Pseudomonas aeruginosa* infection is defined as P. aeruginosa in ≥50% of the swabs or at least two of three documented *Pseudomonas*-specific antibody titers above the threshold of 1:1250.

### Blood samples

Blood draws take place in case of routine blood sampling for clinical purposes. At least annually, basic parameters such as blood gas analysis to objectify gas exchange and blood count and parameters for systemic inflammation are determined. Levels of fat-soluble vitamins are checked on a yearly basis to avoid deficiencies by optimized supplementation. Additionally, liver, renal, pancreatic function are examined due to the potential affection of these organs in children with CF.

### Biobanking

A complementary biobank was set up simultaneously. Samples from study participants are collected following the parents' or legal guardians' consent. Microbiological nose and throat swabs and sputum samples are collected regularly and excess material as well as tissue from intervention or operation are frozen for future analysis. Serum tubes are taken for protein preservation, EDTA and Paxgene tubes are used for DNA and RNA storage. The material is securely stored in a freezer at −20 to −80°C, as appropriate, at each participating site.

### Multiple-breath washout

Multiple-breath washout testing is performed with the Exhalyzer D system (Eco Medics, Duernten, Switzerland) and weight- and age-adapted protocols using either 4% sulfur hexafluoride (SF_6_) or resident nitrogen (N_2_) as tracer gas as previously described (53, 54). All MBW measurements are (re-)analyzed using Spiroware version 3.3.1 (Eco Medics, Duernten, Switzerland) (55). The main parameter derived from MBW is the LCI_2.5_, reflecting the number of lung turnovers necessary to clear a tracer gas from the lungs with higher values indicating more ventilation inhomogeneity. For age- and tracer gas-overarching analyses, LCI_2.5_ values are transformed into z-scores, and the upper limit of normal is determined based on control groups of children without lung disease examined under the same conditions with SF_6_- or N_2_-MBW, respectively (56).

### Morpho-functional chest MRI

Annual MRI scans are performed in all study participants as previously reported (36, 57). Infants and children under the age of 6 years undergo sedation with chloral hydrate for this procedure. Children older than 6 years are capable of performing the MRI procedure without any medication. Chloral hydrate is preferred compared to other sedatives to enable free breathing and to reduce sedative-induced atelectasis (58, 59). In short, T1-weighted sequences before and after intravenous contrast, T2-weighted sequences, and first-pass perfusion imaging are acquired in free breathing using a clinical 1.5T MR scanner (Magnetom Avanto, Siemens Medical AG, Erlangen, Germany). Images are assessed for morphological abnormalities (MRI morphology score and subscores for wall thickening/bronchiectasis, mucus plugging, sacculations/abscesses, consolidations, and pleural reaction) and perfusion deficits using a validated MRI score as previously described (36, 60). Perfusion studies with administration of intravenous contrast material were limited to children ≥12 months of age before its approval for use in infants in Germany in 2015 (61). The extent of structural abnormalities and abnormal perfusion are rated in each lobe as 0 (no abnormality), 1 (<50% of the lobe involved), or 2 (≥50% of the lobe involved). The global score reflects the sum of the morphology and perfusion score. An MRI score > 0 is defined as abnormal (62).

### Quality of life questionnaire CFQ-R

To examine personal and mental well-being as important component of CF treatment, the health-related quality of life is evaluated using age-specific versions of the disease-specific and validated Cystic Fibrosis Questionnaire-Revised (CFQ-R) (63, 64). It comprises three categories: health-related QoL, symptoms and overall health perception. Each category is rated by the child from the age of 6 years and/or its parents. The grading follows the four levels according to the Likert-scale.

### Study endpoints and objectives

Primary study endpoints are the LCI_2.5_ and the MRI score. Secondary endpoints are thriving, the number of PEx, the rate of detection of proinflammatory pathogens and the CFQ-R. *CFTR* genotypes, the presence of symptoms such as cough, and the use of CFTR modulators will be part of an explorative analysis on risk and protective factors for disease progression. The main objective of this TRACK-CF study is to identify risk factors and protective factors that determine the onset and progression of CF lung disease (Figure 1). Subaims are i) the implementation and validation of MBW and MRI as sensitive non-invasive outcome measure, ii) cross-sectional and longitudinal evaluation of MBW and MRI, their mutual relationship and differences between the 3 diagnostic groups to characterize disease progression, severity and response to therapy, iii) the association of MBW and MRI with clinical and microbiological data to identify protective and risk factors such as the role of environmental factors, PEx and pathogens and iv) the analysis of serum markers and the genome in order to identify new biomarkers and modifier genes. Additional research questions may arise during the course of the study.

### Data management

Source data of the standardized questionnaires and visit checklists are transferred to a Microsoft Access data base (Access 2016, Microsoft Corporation, Redmond, WA, USA) and can be extracted and analyzed with SPSS Statistics version 22 (SPSS Inc., Chicago, IL, USA) and R (R Core Team 2016, R foundation for Statistical Computing, Vienna, Austria) according to the respective scientific question and study endpoint.

## Discussion

TRACK-CF is a unique prospective longitudinal observational trial including pediatric patients with CF from diagnosis onwards aiming at an enhanced investigation and understanding of early CF lung disease. TRACK-CF is a comprehensive approach combining a deeply phenotyped patient cohort with the collection of biomaterials. Furthermore, it is the first observational study on early CF lung disease with sensitive endpoints of morpho-functional changes recruiting both children diagnosed by CF NBS and based on clinical symptoms side by side without the need for historical controls.

Several other ongoing observational cohort studies such as the London Cystic Fibrosis Collaboration (LCFC), the Australian Respiratory Early Surveillance Team for CF (AREST CF) and the Study of Host Immunity and Early Lung Disease in CF (SHIELD CF) focus on early CF lung disease and collect clinical parameters, microbiological data, lung function testing and imaging methods during childhood (34, 65, 66). So far, CT has been the gold standard for thoracic imaging in CF and has shown the occurrence of first structural changes in the CF lung at an age of three months underlining the relevance of imaging tools (10). Despite its decreased spatial resolution compared to CT, chest MRI has gained importance due to its high sensitivity to detect morpho-functional changes. Of note, perfusion abnormalities visualize hypoxic pulmonary vasoconstriction due to mucus plugging including airways that are not visible by MRI adding to the value of MRI in imaging CF lung disease. Compared to AREST CF and LCFC using CT, the broad implementation of MRI as morpho-functional and radiation-free method is novel in the TRACK-CF cohort (67).

The regional introduction of the NBS in Germany has brought the exceptional opportunity of simultaneous groups of children with CF at the same age, but with different modes of diagnoses, i.e., either following NBS or clinical signs and symptoms typical for CF. In other countries, the NBS is immediately implemented nationally which consequently leads to a divergent observation period in older, clinically diagnosed children and younger newborn screened children. Bearing in mind the regular adaption of CF therapy, the parallel examination of all children at the same age prevents confounding results due to shifted study periods and historic control groups. This is important given the tremendous improvements in therapy and life expectancy in patients with CF in the last decades.

The TRACK-CF cohort has been initiated in 2011 and has grown ever since. So far, 163 children have been recruited and have been followed for up to 10 consecutive years. To enlarge the understanding of early CF lung disease, appropriate non-invasive outcome measures suitable for the use in infants and preschool children are necessary. The initial trial phase has therefore focused on the establishment and validation of the applied methods, especially MBW as lung function and MRI as a morpho-functional modality. The implementation of MBW in the TRACK-CF cohort has proven its feasibility in the real-life setting (68). Analysis of MBW in sedated infants and preschool children below the age of 4 years across multiple centers resulted in about 90% of successful measurements with satisfying comparability (68). In experienced centers and those naïve to the performance of MBW, an overall success rate of 82.4% was achieved in awake children at preschool age (69). The sensitivity of MBW to detect ventilation inhomogeneities in children with CF and other lung diseases was shown to be high across participating centers (68, 70). The analysis of the mean LCI_2.5_ showed a significantly higher LCI_2.5_ in children with CF compared to age-matched healthy controls with a significantly higher frequency of elevated LCI_2.5_ in affected children (68–70). Other studies have described a larger individual variability of LCI_2.5_ following intravenous treatment of PEx (71, 72) than our results (56, 68–70). The difference might be explained by the younger TRACK-CF age range in which mobilization of mucus may lead to an increased ventilation homogeneity compared to a recruitment of initially obstructed airways in older children and adults.

Routine chest imaging was previously based on CT. Comparison of CT and MRI found similar sensitivity to detect morphological changes and an advantage of MRI to evaluate functional properties through perfusion analysis (37). The TRACK-CF cohort has significantly advanced the introduction and validation of MRI as non-invasive outcome measure in children. In a cross-sectional analysis, morpho-functional alterations in children with CF, especially wall thickening and mucus plugging, were more frequent and more severe compared to non-CF lung disease controls (60). Therapy of PEx resulted in a decrease of MRI scores in children with CF reflecting treatment response (60). MRI was successfully implemented across four CF centers of the German Center for Lung Research (DZL), including the development of a standardized protocol (62). Recently, MRI delineated the longitudinal progression of early CF lung disease in early childhood (73).

To analyze the relationship of abnormalities detected by the two methods, MBW and MRI were compared (56). MBW and MRI showed a good correlation in clinically stable patients with CF spanning the entire pediatric age range (74). Both LCI_2.5_ and MRI scores allowed a differentiation between children with CF with mild and those with more advanced lung disease (60, 74). Both outcome measures were able to detect improvement after antibiotic therapy of a PEx underlining their complementary benefits. The TRACK-CF study also served for follow-up visits of the randomized, double-blind, controlled study on the preventive inhalation of hypertonic saline in infants with CF (PRESIS), which demonstrated a reduction of the LCI_2.5_ but no difference in MRI morphology scores and frequency of PEx (75). The TRACK-CF study was furthermore the first study to capture a positive impact of NBS on morpho-functional changes in the lung during the first years of life. Despite effective strategies to decelerate clinical disease development and widely achieved clinical stability in early childhood, examinations by chest MRI demonstrated progression of morpho-functional alterations in children with CF (73). Previously, applicable and sensitive methods in young children without the need of active cooperation and breathing maneuvers as well as radiation-free imaging modalities had not been available and validated. The TRACK-CF cohort has already made important contributions to the advancement and validation of MBW as a measure for lung ventilation homogeneity and MRI as method to assess lung morphology and perfusion and has highlighted their complementary benefit (74).

Various studies have demonstrated the persistent progression of early CF lung disease despite clinical stability in childhood (13, 28, 73, 76). The overall good phenotype-genotype correlation on a cohort-level does not sufficiently account for the known phenotypic variability on an individual level, which consequently necessitates the search for CFTR modifier genes, non-genetic and environmental risk factors (41). Pulmonary exacerbations, bacterial infection with proinflammatory pathogens and respiratory symptoms such as cough have proven aggravating effects on the pulmonary outcome (51, 73). Similarly, environmental factors such as air pollution and secondhand smoke have been described as risk factors for worsening of disease (77, 78). In the face of widely used NBS programs and carrier testing, negative effects of these risk factors on the incidence and prevalence of CF are challenging to quantify (79). The TRACK-CF cohort comprehensively documents such risk factors and their association with pulmonary morpho-functional alterations and blood biomarkers based on DNA, RNA and proteins and metabolites, providing a comprehensive basis for the assessment and quantification of adverse and beneficial effects of individual parameters. Vice versa, the TRACK-CF cohort including children after different modes of diagnosis in a concurrent setting permits quantification of the effects of NBS, which were previously reported with historic controls only (14, 80, 81). NBS has however led to an increased number of *CFTR* variants of unknown clinical significance which highlights the use of predictive biomarkers for adequate follow-up. Biomarkers of disease severity, progression and response to therapy hold promise to optimize patient care including individualized therapeutic approaches (15, 82). Moreover, the emergence of highly-effective targeted therapies such as CFTR modulators has changed the prospects of patients with CF (83). Highly-effective targeted therapies are increasingly used in children and the comprehensive TRACK-CF study offers decisive information on their impact on early CF lung disease.

The search for modifier genes and the gene expression by blood transcriptomic analysis can be combined with the recorded clinical, microbiological and morphofunctional data of participating children. The multi-omics approach aims to close the gap between genotype and phenotypic presentation since both, genetic and non-genetic factors, influence the onset, course and severity of CF lung disease. Individual analysis of blood transcriptomic, proteomic and metabolomic signatures have shown promising results to detect treatment response and association with pulmonary pathologies in CT in older children and adults with CF (84, 85). The complex interplay between mucus, inflammation and infection precedes functional and structural lung damage and will be addressed by the longitudinal evaluation. TRACK-CF may substantially contribute to the identification of relevant pathways linking impaired mucociliary clearance with inflammatory processes and host-microbiota interaction (86, 87). Furthermore, associations between known risk factors such as PEx with ventilation inhomogeneities and CF-typical changes such as bronchial wall thickening and mucus plugging detected by MBW and MRI will be assessed (88). As MRI provides detailed information on the characteristic abnormality and localization in the CF lung, it can be leveraged for system medicine approaches. Finally, understanding underlying molecular mechanisms and identifying relevant molecules facilitates the development of novel therapeutics to improve and advance CF care.

In summary, the comprehensive data collection, the innovative and novel examination approaches including MBW and MRI and the unique composition of participants in the TRACK-CF cohort offers information on risk and protective factors for the development and course of early CF lung disease. It offers relevant non-invasive study endpoints and provides valuable insights into the disease onset and progression. TRACK-CF has the potential to answer urgent questions regarding the development, risk and protective factors, biomarkers and novel therapeutic approaches for the progression and prediction of early CF lung disease.

## Ethics statement

The studies involving human participants were reviewed and approved by the Ethics Committees of the University of Heidelberg (approval S-211/2011) and each participating site and is registered at clinicaltrials.gov (NCT02270476). Written informed consent to participate in this study was provided by the participants' legal guardian/next of kin.

## Author contributions

ES: conceptualization, formal analysis, investigation, and writing—original draft. OS: conceptualization, formal analysis, investigation, and writing. SG: conceptualization, investigation, and writing—review and editing. CJ, CL, GN, IRi, and IRu: investigation and writing—review and editing. MK: investigation, writing—review and editing, and funding acquisition. A-MD: data acquisition and review and editing. MM: conceptualization, writing—review and editing, supervision, and funding acquisition. MS: conceptualization, investigation, writing—review and editing, supervision, and funding acquisition. All authors contributed to the article and approved the submitted version.
